# Self-Trephination Resulting in Exposed Brain Matter and Cerebral Abscess in a Schizophrenic Patient With Delusions

**DOI:** 10.7759/cureus.77003

**Published:** 2025-01-06

**Authors:** Dipesh Bhatt, Elliot Sanchez, Taleah Angus, Karen Li, Murat Cosar

**Affiliations:** 1 Department of Surgery, Nassau University Medical Center, East Meadow, USA; 2 Department of Neurosciences, Nassau University Medical Center, East Meadow, USA

**Keywords:** foreign body delusions, schizophrenia, self-inflicted brain injury, self-inflicted injury, self-trephination, traumatic brain injury

## Abstract

A 56-year-old woman with schizophrenia presented with persistent foreign body delusions, who chronically excavated her scalp and skull, resulting in a large cranial wound with granulation tissue, purulent discharge, and pneumocephalus. CT scans revealed bilateral subdural hematomas (SDH), polymicrobial osteomyelitis, and a brain abscess causing cerebral edema. The neurosurgical intervention involved cranial washout, debridement, and scalp reconstruction via rotational flap closure, followed by broad-spectrum antibiotics tailored to culture results. Hyperbaric oxygen therapy (HBOT) was employed to promote faster healing and lower postoperative complications and potential reinfection. Psychiatric stabilization with antipsychotics addressed medication noncompliance, improving the patient’s engagement with care. Postoperatively, she regained functional independence and was transitioned to inpatient rehabilitation for mobility and activities of daily living. This case highlights the critical role of integrated care involving neurosurgery, psychiatry, infectious disease, and rehabilitation in managing rare and complex presentations of self-trephination. HBOT is noted for its efficacy in promoting healing under challenging conditions.

## Introduction

Trephination, as a surgical procedure, is most notable for its historical importance as one of the earliest known surgical practices [1]. It is almost inconceivable to encounter such cases in the 21st century. Nevertheless, we are presenting a case of a schizophrenic female patient with delusions of foreign objects pervading her skull who chronically picked at her skull until it resulted in an open cranial wound exposing the brain. This case is one of the very few documented cases of extreme self-harm leading to open head injury with exposed brain matter and cerebral abscess caused by underlying schizophrenia.

Schizophrenia is a chronic mental disorder characterized by alterations in perceptions, persistent and possibly disturbing delusions, as well as occasional neurocognitive impairment [2]. Self-harming behaviors have been noted with this condition which commonly include head-banging reckless behavior leading to self-harm and suicide. Our schizophrenic patient presented differently, she had been chronically digging into her skull to stop foreign bodies that she believed were invading her skull, leading to an open injury of the skull exposing the brain matter superimposed on osteomyelitis and brain abscess with cerebral edema [3].

## Case presentation

**Figure 1:**
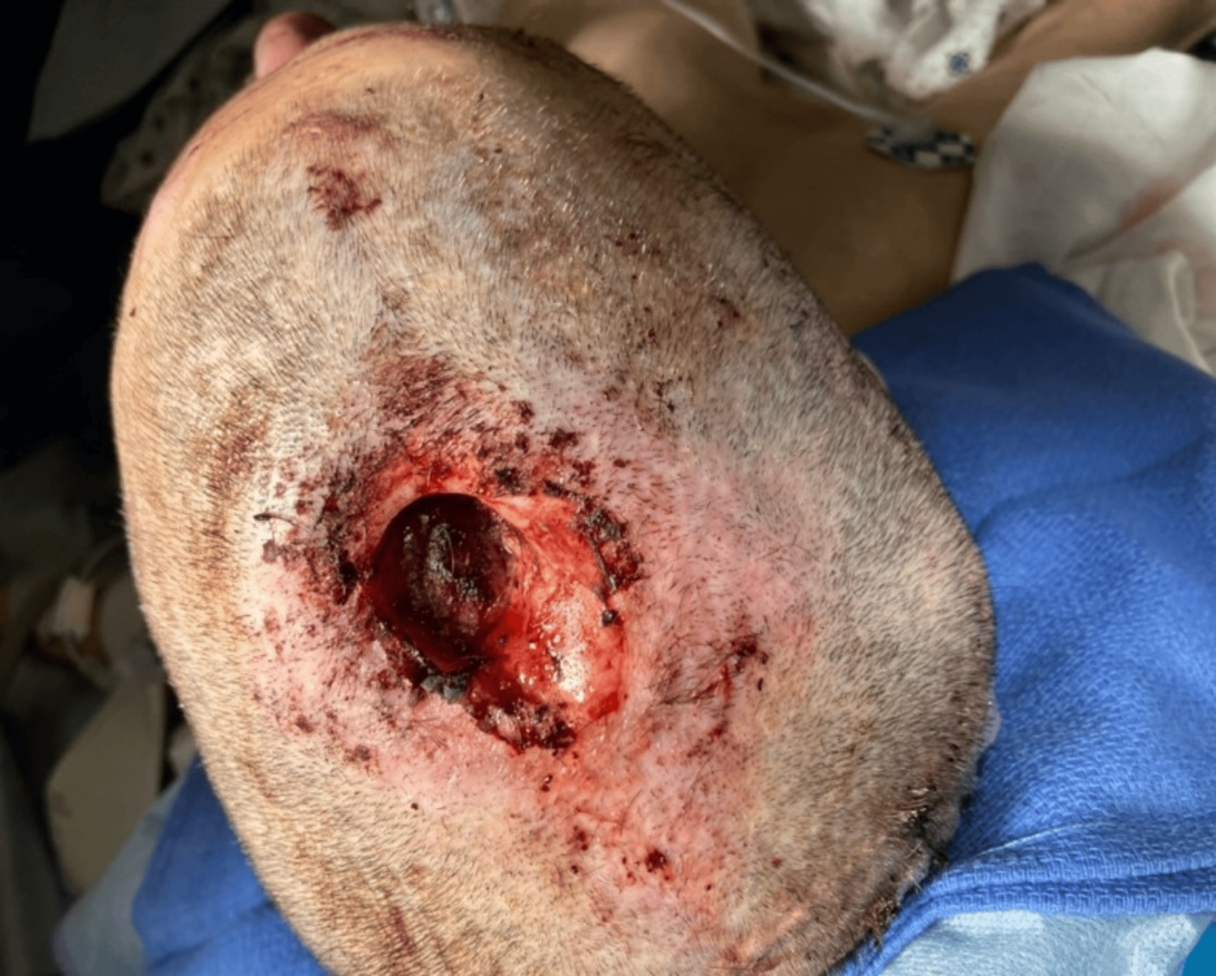
Gross appearance of the patients' wound on initial presentation

A 56-year-old female patient with a history of schizophrenia with foreign body delusions presented with a large head wound and exposed brain matter. Collateral history obtained from her sister states she would feel a sensation of foreign bodies crawling inside her skull, and she tried to remove the sensation by repeatedly burrowing into her skull using her finger. Her Glasgow Coma Scale (GCS) score was 11 (best eye response (E): 4/best verbal response (V): 1/best motor response (M): 6) and with stable vital signs. The head wound measured 4 x 3 x 4 cm on the apex of the scalp (Figure 1), was not actively bleeding, and had chronic features with granulation tissue and some purulent discharge. 

**Figure 2:**
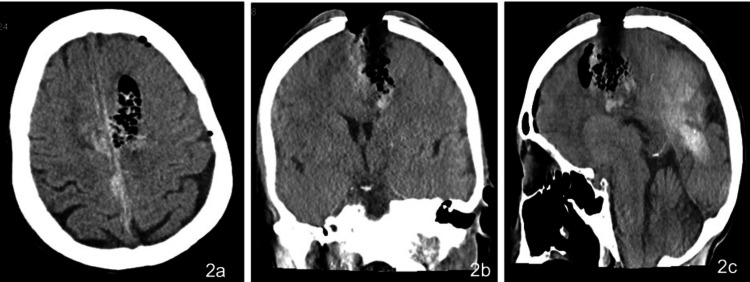
Preoperative (a) axial, (b) coronal, and (c) sagittal plane CT showing open brain injury with surrounding pneumocephalus and foci of infection suggesting cerebral abscess

Laboratory studies done at the time of admission showed mild leukocytosis with low hemoglobin and RBC counts (Table 1). CT scans revealed bilateral parafalcine subdural hematomas (SDH), intraparenchymal mixed hemorrhages (IPH), pneumocephalus, and polymicrobial skull osteomyelitis with brain abscess causing cerebral edema (Figures 2a-2c).

**Table 1:** Complete blood count (CBC) on admission, after the initial washout and flap closure, atter the second washout, and before discharge WBC: white blood cell

	Admission	After initial washout and flap closure	Post-second washout	Before discharge from the hospital
WBC (4500-11000)	14330	5400	5600	3990
Hemoglobin (12-16 g/dL)	6 g/dL	9.3 g/dL	9.4 g/dL	10.7 g/dL
Hematocrit (38-47%)	22%	30%	28.3%	33.2%
Neutrophils (38.1-78.1%)	96%	79.6%	---	79%

A cranial washout and debridement of the infected tissues including the brain, followed by prompt closure with a rotational flap of the scalp, was performed to clean out and close the affected area. Postoperatively, she was started on intravenous (IV) meropenem, vancomycin, and metronidazole after wound cultures grew MSSA (methicillin-sensitive *Staphylococcus aureus*), pan-susceptible *Escherichia coli, Pseudomonas, and Klebsiella*. Following the surgery, she was minimally responsive, often choosing to ignore female members of the healthcare team and only minimally speaking to male members of the team. This contrasted with her presentation previously, where she was self-aware and logically verbal, and contributed to her combination of sedating psychiatric medications and possible catatonia.

**Figure 3:**
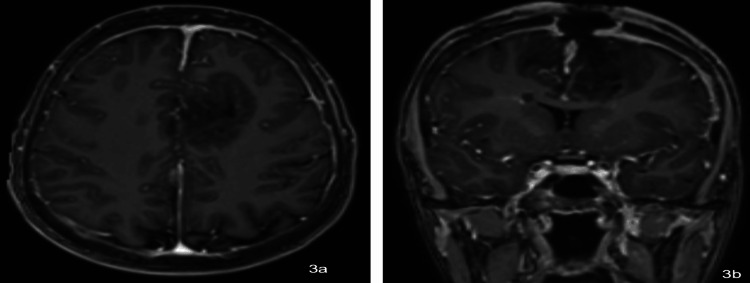
Postoperative day 1 MRI in (a) axial and (b) coronal planes showing slight pneumocephalus and cerebral edema without midline shift

**Figure 4:**
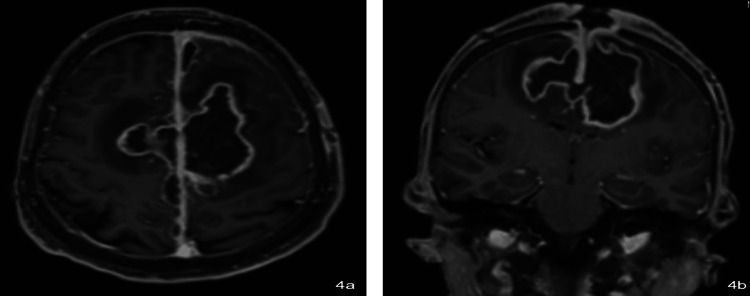
Postoperative day 10 MRI in (a) axial and (b) coronal planes showing bifrontal parafalcine abscess collection with surrounding edema and hemorrhage

Early postoperative day 1 MRI (Figures 3a-3b) showed changes including pneumocephalus and edema. The patient continued with planned broad-spectrum antibiotic regimens. Her second MRI on postoperative day 10 (Figures 4a-4b) showed a bifrontal parafalcine abscess with edema and hemorrhage. She was planned to undergo repeat washout and debridement of the abscess and continued to be medically managed with broad-spectrum antibiotics. The contrasted brain MRI two weeks after the second washout showed decreased dimensions of the abscess.

**Figure 5:**
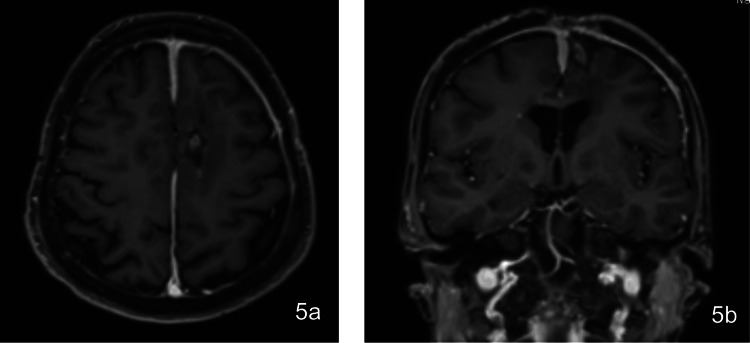
Postoperative MRI at two months in the (a) axial and (b) coronal planes showing closed calvarium with near resolution of cerebral abscess

She also received hyperbaric oxygen therapy (HBOT) after the second washout because of the concerns for the scalp healing which continued for six weeks. After the completion of hyperbaric therapy and broad-spectrum antibiotic regimens, the follow-up brain MRI (Figures 5a-5b) two months after the second washout showed a near-complete resolution of the brain abscess. Tables 1-2 demonstrate the patient’s progress, with improvements in white blood cell (WBC) count, hematocrit, hemoglobin, electrolyte stabilization, and blood urea nitrogen (BUN) levels from admission to discharge.

**Table 2:** Complete metabolic panel (CMP) with electrolytes and kidney function test on admission, after the initial washout and flap closure, atter the second washout, and before discharge BUN: blood urea nitrogen

	Admission	After initial washout and flap closure	Post-second washout	Before discharge from the hospital
Sodium (136-146 mEq/L)	139 mEq/L	143 mEq/L	137 mEq/L	139 mEq/L
Potassium (3.5-5.0 mEq/L)	4.3 mEq/L	4.4 mEq/L	4.3 mEq/L	4.2 mEq/L
Chloride (98-107 mEq/L)	106 mEq/L	106 mEq/L	104 mEq/L	107 mEq/L
Bicarbonate (22-28 mEq/L)	18 mEq/L	28 mEq/L	27 mEq/L	26 mEq/L
Anion gap (5-15)	15	9	6	6
BUN (9-23 mg/dL)	43 mg/dL	38 mg/dL	18 mg/dL	16 mg/dL
Creatinine (0.6-1.0 mg/dL)	0.7 mg/dL	<0.5 mg/dL	0.6 mg/dL	0.5 mg/dL

During the duration of her hospitalization, she was also started on antipsychotic medicines given her history of medication noncompliance for her schizophrenia. Postoperatively, there were concerns about her possibly becoming catatonic as she was not responding to verbal cues and refused to speak to or respond to the healthcare team; however, after administration of her antipsychotic medications, she became more responsive and verbal and was ambulating unassisted with a GCS of 15 near the end of her hospital stay.

**Figure 6:**
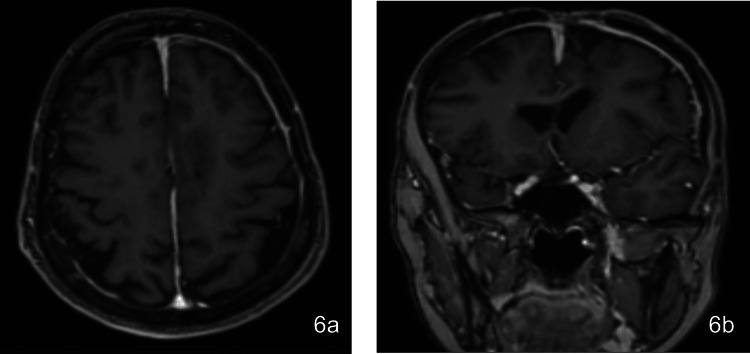
Postoperative MRI at six months in the (a) axial and (b) coronal planes six months posthospital discharge showing resolution of abscess and healing of the closed calvarium

Due to limitations in mobility, gait, balance, and ability to perform activities of daily living (ADLs) independently, she was evaluated by physical medicine and rehabilitation (PM&R) and eventually transferred to their care to improve her functionality and found to be an appropriate candidate for acute inpatient rehabilitation. Plans were made for her transfer to a long-term care facility after medical clearance. Psychiatric support was continued throughout, managing her psychiatric symptoms and ensuring her safety and appropriate care decisions. Resolution of abscess and evidence of healing in the closed calvarium can be seen in the postoperative MRI findings obtained six months after hospital discharge (Figures 6a-6b).

## Discussion

Traumatic brain injuries of psychiatric patients resulting in open wounds to the brain matter are not common, and cases of self-trephination are even less commonplace. A recent report documented five cases in a four-year period, highlighting the rarity of self-trephination in the English literature. While this could reflect the true incidence, it is also possible that some cases remain undocumented due to underreporting or misclassification [4]. Treatment of such injuries poses concerns including preoperative stabilization of the patient, surgical complications, and postoperative critical care [5].

Our patient's wound to the calvarium primarily required urgent neurosurgical intervention given the exposed brain tissue and subsequent osteomyelitis with brain abscess. Elimination of the primary foci of infection is paramount to ensure optimal patient outcomes and must be followed with prompt intervention with intravenous antibiotics for 4-6 weeks for a single focus of brain abscess [6]. Cranial washout and debridement of the affected area helped eliminate the primary nidus of infection and minimized the event of recurrence.

Empiric antibiotic therapy postoperatively was also a key component of this patient's postsurgical treatment. A "triple high-dose" therapy for six weeks is the recommended therapy as per the literature and should be conducted with antibiotics with adequate cerebrospinal permeability. A combination of meropenem/imipenem, cefotaxime, and metronidazole intravenously for two weeks, followed by oral for four weeks is the standard treatment; however, studies also show that carbapenem monotherapy or in conjunction with metronidazole also yields similar patient outcomes as used with our patient [5]. Although steroids could be used and have been shown to reduce cerebral edema postoperatively, many of those findings were in patients with cerebral malignancies and no other mass lesions; the patient's postoperative cerebral edema was not significant or symptomatic and was managed with HBOT instead.

This case represents only the seventh known case of a schizophrenic patient with foreign body delusions performing self-trephination that can be found in the English literature [6] and one of the few cases documented where self-harm is inflicted without the use of tools or objects and without a background of substance abuse [7]. This case also presented a delicate interplay between multidisciplinary teams including neurosurgery and plastics for the debridement and rotational flap closure, psychiatry to manage the patient's schizophrenia, infectious disease to monitor the patient's wound care and recurrent abscess risk, and the ICU for the patient's daily nutrition and activity needs. Management of this patient with this multidisciplinary approach demonstrates what intervention, and when, should be performed by the respective healthcare teams to ensure synergy in the common goal, the patient's overall well-being.

The use of hyperbaric treatment in this case is also noteworthy, given that there was no underlying bone at the site of the surgical procedure meant tissue healing would become complicated and there was a risk of potential reinfection. Literature shows that the use of HBOT in traumatic brain injury (TBI) promotes revascularization, prevents cellular apoptosis, and can suppress inflammatory cytokines that can further damage the site [8]. Hyperbaric treatment in our case allowed the patient's body to the adequate conditions to facilitate healing and provided the body a base upon which to begin the process of bone remodeling and skin healing. Although this method of treatment requires more research, it is a distinctive approach to this rare case.

## Conclusions

In psychiatric patients, self-inflicted traumatic injury should always be kept in mind when they present to a hospital. In this case, self-trephination was treated by a multidisciplinary team with surgical washout and debridement with rotational flap closure, broad-spectrum antibiotics, and antipsychotics. HBOT was used to induce faster healing and lower postoperative complications and potential reinfection, demonstrating its potential contribution to recovery in this rare case.
